# Editorial: Advances of endocrine and metabolic cardiovascular outcomes: From basic to clinical science, volume II

**DOI:** 10.3389/fendo.2022.1114632

**Published:** 2023-01-12

**Authors:** Wenzhuo Cheng, Ye Ding, Chengqi Xu, Qiulun Lu, Si Jin

**Affiliations:** ^1^ Department of Endocrinology, Institute of Geriatric Medicine, Liyuan Hospital, Tongji Medical College, Huazhong University of Science and Technology, Wuhan, China; ^2^ Center for Molecular and Translational Medicine, Georgia State University, Atlanta, GA, United States; ^3^ Key Laboratory of Molecular Biophysics of the Ministry of Education, College of Life Science and Technology and Center for Human Genome Research, Huazhong University of Science and Technology, Wuhan, China; ^4^ Nanjing Medical University, Nanjing, China

**Keywords:** ASCVD, metabolic diseases, diabetes, cardiovascular diseases, endocrine dysfunctions

There is a growing body of research on the pathogenic mechanisms of atherosclerotic cardiovascular diseases (ASCVD), which continues to be the leading cause of mortality worldwide. Endocrine and metabolic diseases, including hyperglycemia, hyperlipidemia, hyperuricemia, hypertension, overweight or obesity, *etc.*, are the main risk factors for ASCVD (1, 2). Meanwhile, more and more anti-diabetic medicines, such as sodium-glucose transporter-2 inhibitors (SGLT2i), glucagon-like peptide-1 receptor agonists (GLP-1RAs), etc., are shown to have direct cardiovascular preventive benefits in addition to decreasing blood glucose levels, which were proved by the significant reduction in 3p MACE risks, independent of glucose-lowering glucose lowering effects. However, the specific regulatory mechanisms of endocrine and metabolic disorders affecting the occurrence and development of ASCVD are not fully understood. Therefore, there is an urgent need to study the pathogenesis of cardiovascular events associated with metabolic disorders in order to develop more effective therapies aimed at preventing and slowing down disease progression. In order to better value the cardiovascular results in the treatment of endocrine and metabolic diseases, this research topic (Volume II) aims to gather current developments emphasizing in this field, both in terms of basic and clinical aspects. We have collected 9 high-quality studies covering recent, novel, clinical significance in endocrine and metabolic cardiovascular diseases in our current research topics.

Diabetic cardiomyopathy (DCM) is a serious complication of diabetes mellitus and is the leading cause of cardiac death in diabetic patients. Therefore, it is crucial to explore the pathogenesis of DCM and find new effective therapeutic targets. Ferroptosis, a novel mode of cell death, is not fully understood in the pathogenesis of DCM. Du et al. obtained DCM model by intraperitoneal injection of streptozotocin in C57BL6 mice. Subsequently, the DCM mice were divided into two groups by the canagliflozin intervention and key aspects relevant to ferroptosis, such as total iron, Fe^2+^, transferrin receptor 1 (TfR1), ferritin heavy-chain (FTN-H), were investigated *in vivo* and *in vitro*. They found that canagliflozin attenuates the development of DCM by inhibiting ferroptosis. Through their research, we learned that ferroptosis may be involved in the process of DCM and prevention of ferroptosis may be effective in delaying the process of DCM. Moreover, Peng et al. focused on whether endothelial cell dysfunction is associated with DCM. The previous research discovered that an endogenous S-nitrosothiol produced by eNOS called S-nitroso-L-cysteine (CSNO), may be connected to the insulin signaling pathway. In the current study, they demonstrate that CSNO treatment promotes glucose uptake by activating the insulin signaling pathway, glucose transporter 4 (GLUT4) membrane translocation. CSNO mitigates cardiomyocyte injury by reducing oxidative stress, autophagy hyperactivation and mitochondrial damage. The data from these studies will improve our understanding of how DCM occurs and offer novel therapy alternatives.

With the in-depth study of the pathogenesis of diabetes, more and more glucose-lowering drugs are used in the clinic. However, it has been a great challenge to explore therapies with good efficacy and few side effects for the overall benefit of patients. Through clinical trials, Wu et al. recruited 20 diabetic patients and 175 normal controls to assess short-term and long-term glycemic and cardiovascular benefits after washed microbiota transplantation (WMT) treatment and they identify that WMT not only improves short- and long-term blood glucose levels, but also has a more significant cardiovascular benefit. Effective glucose lowering by WMT may provide a new means for the treatment of diabetes, which requires more and more in-depth mechanistic studies to provide a theoretical basis.

It is well known that nitric oxide (NO) is strongly associated with the development of vascular disease (3). However, in addition to NO, there are many other gas molecules in the blood that contribute to vascular disease, and research in this area is relatively lacking. A review by Zhu et al. systematically summarizes hydrogen sulfide (H_2_S) in many diseases such as hypertension, atherosclerosis, inflammation, and angiogenesis. Notably, focusing on crosstalk between different gas transmitters in the control of vascular diseases contributes to our better understanding of the disease and to the development of new therapeutic approaches.

The development of disease prevention and treatment strategies depends on the identification of novel biomarkers for CVDs. By examining the expression of NIMA-related kinase 6 (NEK6) in head and neck squamous cell carcinoma (HNSCC), Yang et al. found that NEK6 upregulation in HNSCC. Analysis of the results by integrating several databases showed that NEK6 is mainly involved in the metabolism of extracellular matrix and EMT processes. The level of immune cell infiltration and the expression of different immunological checkpoints were correlated with an increase in NEK6 expression. Therefore, NEK6 may be a potential prognostic indicator and indicate how patients with HNSCC will respond to immunotherapy. Through the data analysis of several databases and verification by qRT-PCR, Wang et al. successfully identify that potential genes connected to pyroptosis that may contribute to the development of diabetic retinopathy are predicted, along with the lncRNAs and miRNAs that are linked to these genes. It is worth noting that the database analysis should be used as a screening method and the screened molecules should be further experimentally validated.

Melatonin (MT) is an important endogenous neuroendocrine hormone that is involved in a variety of physiological functions. Wang et al. identify that MT therapy could reduce the excitotoxicity model caused by glutamate in R28 cells as well as the retinal damage induced by N-methyl-D-aspartic acid (NMDA) in mice. Mechanistically, MT reduces ROS levels to achieve cytoprotective effects, and this process may be regulated by PI3K-AKT and JAK-STAT signaling pathways. This study provides us with a deeper understanding of the pathogenesis of glaucoma and offers new ideas for its treatment.

Finally, in recent years, there has been a growing number of studies on new antidiabetics, of which the most popular are SGLT2i, GLP-1RA and dipeptidyl peptidase-4 inhibitor (DPP-4). With the development of the economy, Li et al. found that there was a significant increase in the number of SGLT2i and GLP-1RA prescriptions in China from 2018 to 2021, and the preference for SGLIT2i over GLP-1RA is correlated with some patient socioeconomic and prescriber factors. Age, gender, and socioeconomic situation can all have an impact on the choice of glucose-lowering medications. Through Meta analysis, Pan et al. identified that compared with DPP4 inhibitor, higher exposure to GLP-1RAs may contribute to the incretin-based treatments’ cardiovascular advantages. These studies will help people with diabetes choose the right medication for them.

## Conclusion and prospects

The current research subject covers the recent advancements in the study of endocrine and metabolic cardiovascular disease, including the new clinical trial index and the unique molecular regulation of basic research. Based on these findings, a more comprehensive understanding of endocrine and metabolic cardiovascular diseases can be achieved to provide mechanical insights in the pathogenesis and pathophysiology of ASCVD formation and more potential therapeutic targets may be identified. Regarding the close relationship between endocrine or metabolic disorders and downstream cardiovascular outcomes, a call for a new subspeciality in internal medicine, cardiometabolic or metabolic cardiovascular medicine, is indeed crucial in the future.

## Author contributions

All authors listed, have reviewed and edited the manuscript, and approved it for publication.
